# Telemedicine‐based diagnosis and management of ocular chemical injury in a remote setting: A case report

**DOI:** 10.1002/jgf2.70017

**Published:** 2025-04-18

**Authors:** Eisuke Shimizu, Hiroki Nishimura, Rohan Jeetendra Khemlani, Shintaro Nakayama, Keitaro Suzuki, Katsuya Sato

**Affiliations:** ^1^ OUI Inc. Tokyo Japan; ^2^ Yokohama Keiai Eye Clinic Kanagawa Japan; ^3^ Sarufutsu Village National Health Insurance Hospital Hokkaido Japan

**Keywords:** ophthalmology, referral, Smart Eye Camera, telemedicine, teleophthalmology

## Abstract

Ocular chemical injuries require immediate diagnosis and treatment, posing challenges in remote regions. We report a case of mild chemical injury caused by an alkali chemical injury, successfully diagnosed and managed using telemedicine. A primary care physician utilized a smartphone‐based anterior segment imaging system (Smart Eye Camera), allowing an ophthalmologist to remotely evaluate and classify the injury as Roper‐Hall grade. Conservative treatment with irrigation and topical medications led to symptom resolution within 1 week. This case highlights the effectiveness of teleophthalmology, suggesting its significant potential for improving ocular care accessibility and timely intervention in medically underserved rural areas.

## 1 BACKGROUND

Ocular chemical injuries are urgent ophthalmic conditions that require rapid diagnosis and treatment to minimize complications.^1^ Especially, alkali agents are lipophilic and thus penetrate ocular tissues more rapidly than acids. They saponify the fatty acids within cell membranes, allowing deeper penetration into the corneal stroma, subsequently leading to the destruction of the proteoglycan ground substance and disruption of collagen bundles. Damaged tissues subsequently release proteolytic enzymes, resulting in additional progressive tissue injury.^2^

Prompt ophthalmological assessment can be challenging in remote and medically underserved areas.^3^ Telemedicine utilizing smartphone‐based devices offers a promising solution for immediate and accurate ocular evaluation, facilitating timely interventions.^4^

Here we report a case of ocular chemical injury that was accurately diagnosed and successfully managed through telemedicine. Informed consent was obtained for the publication of this case report and any accompanying images. A primary care physician in a rural village utilized a teleconsultation system to collaborate with an ophthalmologist, enabling precise diagnosis. This case underscores the potential utility of teleophthalmology in rural or remote settings.

## 2 CASE PRESENTATION

A previously healthy 53‐year‐old woman accidentally instilled an alkaline disinfectant solution (trimethylammonium chloride), typically used for animals, into her right eye, resulting in immediate ocular discomfort and conjunctival redness. She had no significant ocular or systemic medical history.

**FIGURE 1:**
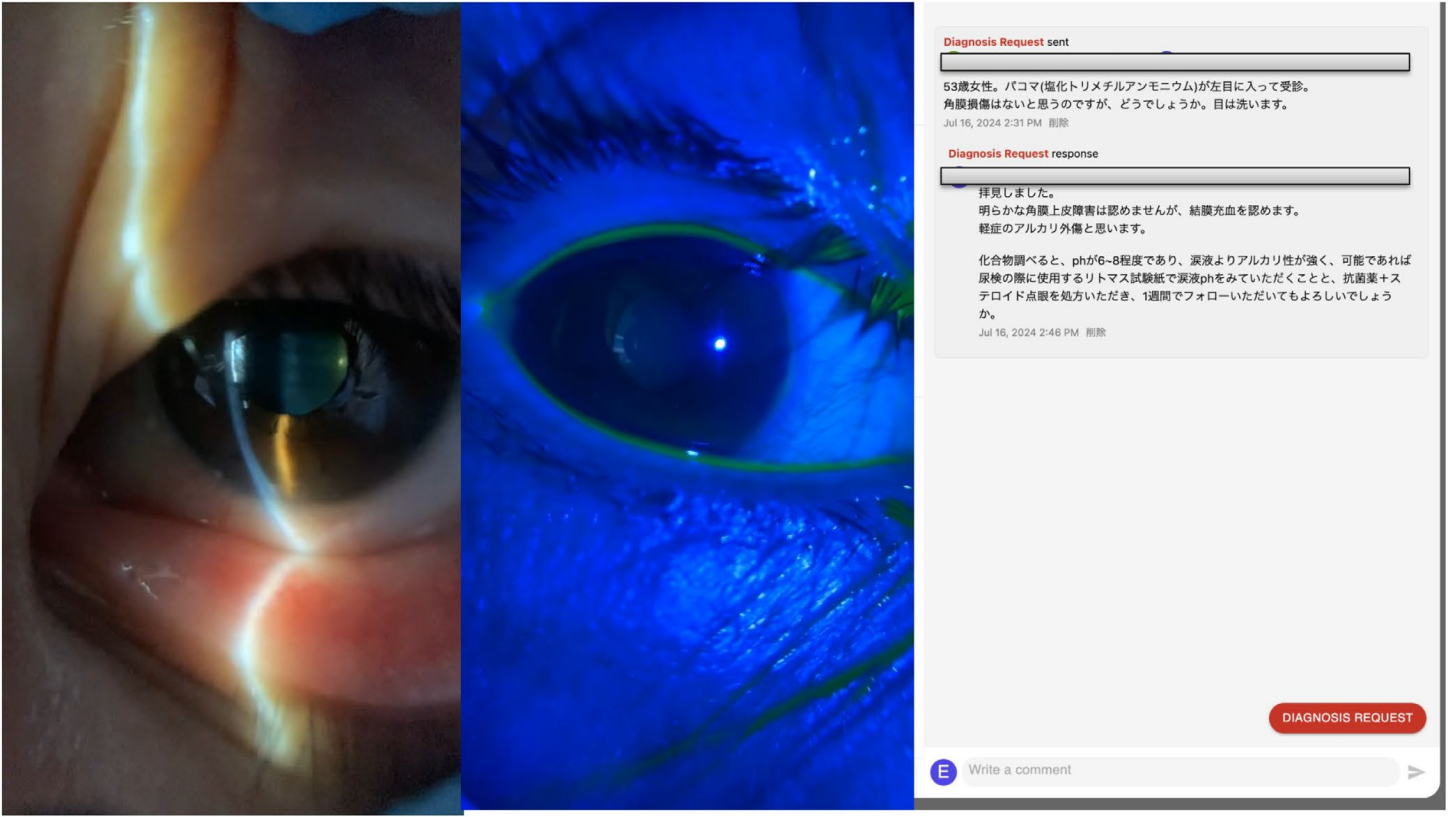
Initial teleophthalmology assessment of chemical eye injury. Anterior segment images captured by a primary care physician using the smartphone‐based portable slit‐lamp microscope. Left panel: Slit‐lamp biomicroscopy shows mild conjunctival hyperemia without corneal limbal damage. Middle panel: Fluorescein staining under blue‐light illumination demonstrates absence of corneal epithelial defects. Right panel: Telemedicine consultation screenshots detailing clinical findings and initial management instructions provided remotely by the ophthalmologist.

Owing to geographical constraints, immediate ophthalmologic evaluation was unavailable, as accessing the nearest ophthalmologist would have required several hours of travel. Therefore, telemedicine was employed, allowing a primary care physician in the patient's village to promptly capture anterior segment images using a smartphone‐based slit‐lamp device (Smart Eye Camera [SEC]). Detailed images were remotely reviewed by an ophthalmologist, who diagnosed the condition as mild chemical injury based on Roper‐Hall classification,^5^ noting the absence of corneal epithelial defects or limbal damage. Based on this remote consultation, immediate ocular irrigation and topical therapy with levofloxacin and fluorometholone eye drops, both administered four times daily, were recommended. Additionally, the pH of the tear film was measured as 7.0 after irrigation (Figure 1).

**FIGURE 2:**
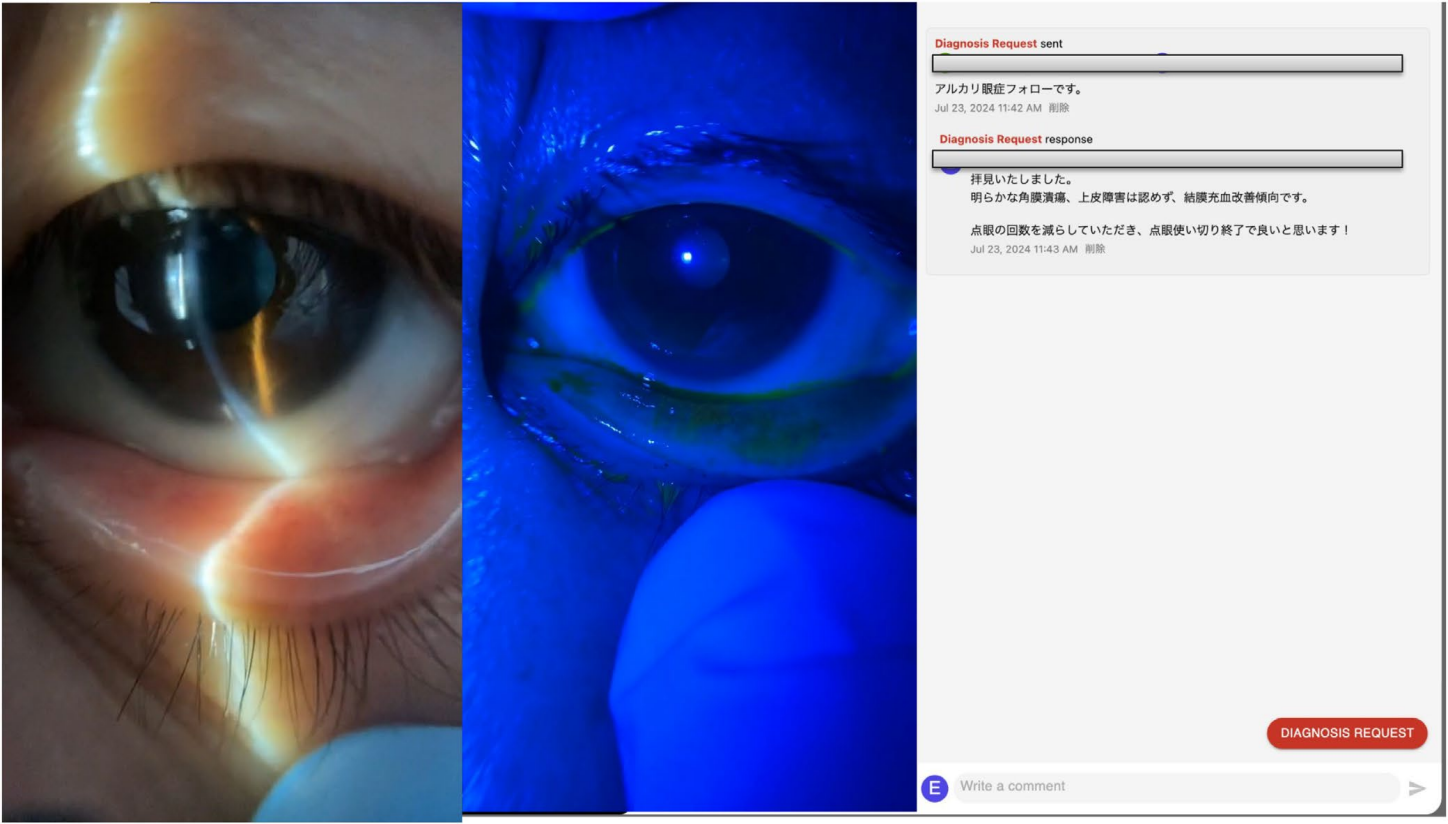
Follow‐up teleophthalmology assessment at 7 days. Anterior segment images taken during follow‐up demonstrate resolution of no conjunctival hyperemia and confirm the absence of corneal epithelial defects or other complications. Remote teleconsultation correspondence confirms clinical improvement, validating the effectiveness of the telemedicine‐guided management plan.

At a 7‐day follow‐up, the patient reported the resolution of subjective symptoms. Follow‐up anterior segment images captured by the primary care physician and reassessed remotely by the ophthalmologist showed marked improvement compared with the initial presentation, confirming the successful resolution of the chemical trauma (Figure 2).

## 3 DISCUSSION

Our case highlights the clinical utility and effectiveness of teleophthalmology, specifically using the SEC, for managing acute ocular injuries in remote areas where access to ophthalmology specialists is limited.

Immediate and thorough ocular irrigation is the most critical initial management step for chemical injuries, aimed at promptly restoring the ocular surface pH to neutral (pH = 7.0).^6^ Subsequent early ophthalmological assessment using slit‐lamp microscopy is essential for accurately evaluating corneal epithelial defects, conjunctival damage, limbal ischemia, and injury severity, typically categorized according to established standards such as the Roper‐Hall classification.^5^

In this case, as the patient's ocular surface pH rapidly normalized after irrigation and the chemical injury was graded as mild (Roper‐Hall grade^1^) via remote anterior segment assessment, conservative management and subsequent telemedicine‐based follow‐up were deemed sufficient, obviating the need for immediate specialist referral.

Previous studies have consistently demonstrated the clinical efficacy of telemedicine combined with portable ophthalmic imaging devices, such as the SEC, across various ophthalmic emergencies, including primary angle closure and traumatic hyphema.^3^, ^4^ The high‐resolution anterior segment images captured by SEC facilitate accurate remote diagnoses and prompt clinical decision‐making.^7^, ^8^ Importantly, teleophthalmology has shown comparable diagnostic accuracy to conventional ophthalmological evaluations, providing an effective solution for improving access to eye care, especially in remote or underserved regions.^9^

However, telemedicine‐based ophthalmology has inherent limitations, including dependency on the quality of acquired images, the technical skills of the user, and the requirement of fluorescein staining, which may not be consistently available in all rural healthcare settings. Overcoming these challenges through targeted training, standardized imaging protocols, and improved resource availability is critical to broadening the application and reliability of teleophthalmology.

Artificial intelligence (AI) holds promise for addressing some of these limitations. Given ophthalmology's significant reliance on diagnostic imaging, AI‐driven algorithms have already been successfully developed for clinical applications, such as determining the necessity and optimal timing for cataract surgery.^10^ Integrating AI with telemedicine technologies can further enhance remote patient screening, facilitate accurate triage, and streamline specialist referrals, ultimately improving clinical efficiency and extending ophthalmic care to broader patient populations.^10^ Once AI enables early and accurate diagnosis, it will become possible to provide more appropriate and timely ophthalmic care for acute ophthalmic conditions, such as the case presented here, by integrating AI‐based diagnostics with telemedicine approaches.

## 4 CONCLUSION

Teleophthalmology using portable smartphone‐based imaging effectively facilitated diagnosis and management of mild ocular chemical injury in a remote setting. Immediate irrigation, remote ophthalmic evaluation, and appropriate topical treatment resulted in rapid resolution without specialist referral.

While image quality and fluorescein staining dependence present challenges, integrating AI could further enhance teleophthalmology's reliability and scope. This approach demonstrates significant potential for addressing urgent ophthalmic conditions in underserved areas, emphasizing the importance of expanding telemedicine infrastructure and training to ensure wider implementation and improved ophthalmic care accessibility.

## AUTHOR CONTRIBUTIONS

**Eisuke Shimizu:** Conceptualization; investigation; writing – original draft; funding acquisition; resources; project administration; visualization; validation. **Hiroki Nishimura:** Writing – review and editing; methodology; software; data curation. **Rohan Jeetendra Khemlani:** Writing – review and editing; data curation; software; methodology. **Shintaro Nakayama:** Formal analysis; validation; investigation; writing – review and editing; funding acquisition. **Keitaro Suzuki:** Writing – review and editing; formal analysis; conceptualization; methodology; investigation. **Katsuya Sato:** Writing – review and editing; supervision; formal analysis; methodology; conceptualization; investigation.

## FUNDING INFORMATION

This study did not receive any specific grant from funding agencies in the public, commercial, or not‐for‐profit sectors.

## CONFLICT OF INTEREST STATEMENT

E.S. is the founder of OUI Inc. and owns stock of OUI Inc. OUI Inc. has the patent for the SEC (the publication of Japanese Patent No. 6627071, Tokyo, Japan). There are no other relevant declarations relating to this patent. The other authors have stated explicitly that there are no conflicts of interest in connection with this article. OUI Inc. did not have any additional role in the study design, data collection and analysis, decision to publish, or preparation of the manuscript.

## ETHICS STATEMENT

Ethical approval statement: This study was waved due to the nature of case report.

Patient consent statement: Written informed consent was obtained from all included patients.

Clinical trial registration: None.

## CONSENT

The authors have obtained patient consent.
